# Automated digital reporting of clinical laboratory information to national public health surveillance systems, results of a EU/EEA survey, 2018

**DOI:** 10.2807/1560-7917.ES.2020.25.39.1900591

**Published:** 2020-10-01

**Authors:** Katrin Claire Leitmeyer, Laura Espinosa, Eeva Kaarina Broberg, Marc Jean Struelens, the ECDC National Focal Points laboratory e-reporting survey group members, ECDC National Focal Points laboratory e-reporting survey group, ECDC National Focal Points laboratory e-reporting survey group, Franz Allerberger, Yves Dupont, Steven Van Gucht, Sophie Quoilin, Iva Christova, Blazenka Hunjak, Christos Karagiannis, Pavla Křížová, Jitka Částková, Eva Møller Nielsen, Jonas Kähler, Rita Peetso, Saara Salmenlinna, Teemu Möttönen, Bruno Coignard, Michaela Diercke, Alkiviadis Vatopoulos, Ákos Tóth, Karl Gustaf Kristinsson, Eleanor McNamara, Annalisa Pantosi, Violeta Mavcutko, Algirdas Griškevičius, Joël Mossong, Christopher Barbara, Titia Kortbeek, Dominique Caugant, Ulf Dahle, Line Vold, Anna Skoczyńska, Jorge Machado, Gabriel Ionescu, Lucia Madarova, Mária Avdičová, Metka Paragi, Julio Moreno Vazquez, Karin Tegmark Wisell, Maria Zambon

**Affiliations:** 1 European Centre for Disease Prevention and Control (ECDC), Stockholm, Sweden; 2 The ECDC National Focal Points laboratory e-reporting survey group members are listed at the end of the article

**Keywords:** automated data reporting, electronic laboratory reporting, clinical microbiology testing, public health surveillance, digital surveillance, early-warning, communicable diseases, antimicrobial resistance

## Abstract

**Background:**

Timely reporting of microbiology test results is essential for infection management. Automated, machine-to-machine (M2M) reporting of diagnostic and antimicrobial resistance (AMR) data from laboratory information management systems (LIMS) to public health agencies improves timeliness and completeness of communicable disease surveillance.

**Aim:**

We surveyed microbiology data reporting practices for national surveillance of EU-notifiable diseases in European Union/European Economic Area (EU/EEA) countries in 2018.

**Methods:**

European Centre for Disease Prevention and Control (ECDC) National Microbiology and Surveillance Focal Points completed a questionnaire on the modalities and scope of clinical microbiology laboratory data reporting.

**Results:**

Complete data were provided for all 30 EU/EEA countries. Clinical laboratories used a LIMS in 28 countries. LIMS data on EU-notifiable diseases and AMR were M2M-reported to the national level in 14 and nine countries, respectively. In the 14 countries, associated demographic data reported allowed the de-duplication of patient reports. In 13 countries, M2M-reported data were used for cluster detection at the national level. M2M laboratory data reporting had been validated against conventional surveillance methods in six countries, and replaced those in five. Barriers to M2M reporting included lack of information technology support and financial incentives.

**Conclusion:**

M2M-reported laboratory data were used for national public health surveillance and alert purposes in nearly half of the EU/EEA countries in 2018. Reported data on infectious diseases and AMR varied in extent and disease coverage across countries and laboratories. Improving automated laboratory-based surveillance will depend on financial and regulatory incentives, and harmonisation of health information and communication systems.

## Introduction

Electronic laboratory-based data reporting has been shown to be an effective and efficient method to automate and improve the timeliness and completeness of communicable diseases and antimicrobial resistance (AMR) surveillance at the healthcare institution level, as well as the national public health level [1-10].

The routine use of digital laboratory data for surveillance purposes has been reported from European countries such as Denmark [11,12] and France [6,13] However, the extent to which such automated laboratory data reporting methods from clinical diagnostic laboratories to national surveillance systems are used has not been mapped systematically in the European Union (EU) and the European Economic Area (EEA).

As a part of the monitoring of EU/EEA national laboratory capacities for public health surveillance and control of infectious diseases, 17 of 30 EU/EEA countries reported, that in 2016, their surveillance networks of clinical laboratories reported microbiology data for at least one disease by machine-to-machine (M2M) upload to a central national public health database [14]. The European Centre for Disease Prevention and Control (ECDC) Public Health Microbiology Strategy [15] foresees that ECDC will, in synergy with relevant European Commission-supported eHealth initiatives, undertake projects to identify and disseminate solutions for automated (M2M) transfer of microbiology data between laboratory and surveillance information systems from the local to national and EU/EEA surveillance levels. The ECDC strategy proposes as target that in 2022, at least 90% of EU/EEA countries will be using automated electronic notification of clinical laboratory data to national surveillance programmes.

The primary objective of this survey was to investigate the modalities and scope of clinical microbiology laboratory data reporting for the national surveillance of EU-notifiable diseases and AMR across Europe. It also assessed the public health use of this information for rapid threat detection and timely response.

## Methods

### Survey tool

The questionnaire administered in the EU survey tool (https://ec.europa.eu/) was developed by ECDC in consultation with the National Microbiology Focal Points (NMFPs) and National Surveillance Focal Points (NSFPs) who are the EU/EEA countries’ designated experts for advice on specific public health functions to ECDC as outlined in the Terms of reference, Annex 3 [16]. NMFPs and NSFPs provided written comments to the first draft of the questionnaire, and carried out a pilot study to check for clarity and usability before launching the survey. Using a Delphi-like approach, two face-to-face discussions were conducted between the study coordinators and survey respondents: the first in October 2018 to revise the draft questionnaire, and the second in June 2019 to critically review and clarify the correct interpretations of terms and definitions used to describe the national reporting systems in order to enhance inter-observer consistency. Furthermore, individual teleconferences were conducted with NMFPs between the two discussions. The final questionnaire included 21 questions supplemented with a glossary of terms.

The questionnaire asked the NMFPs and NSFPs to describe the following items at the national level:

- Use of laboratory information management systems (LIMS) in clinical diagnostic laboratories for reporting test results to clinicians;
- Modes of diagnostic data reporting from clinical diagnostic laboratories to national surveillance databases for the 56 EU-notifiable diseases [17], and for reporting susceptibility data on EU priority indicator antimicrobial-resistant pathogens [18,19] and antiviral-resistant viral pathogens under EU/EEA surveillance (Box);
- Proportion and type of clinical diagnostic laboratories that report data by automated M2M upload from their LIMS to national surveillance system databases (public laboratories, for profit commercial laboratories, non-for profit private laboratories, academic hospital laboratories, national public health microbiology reference laboratories);
- Reasons for non-automated laboratory data reporting to surveillance databases (lack of legal basis/obligation, lack of financial incentive to cover extra cost, lack of information technology (IT) support for data reporting, data protection issues, lack of relevance for surveillance);
- Use of daily or weekly transmitted laboratory data for continuous/frequent cluster event detection and early warning at the national public health level;
- Type and scope of laboratory test data and of patient demographic, clinical and epidemiological data reported on automated basis from LIMS to national surveillance databases;
- Possibility of laboratory data linkage to epidemiological data collected from other sources (e.g. medical case notification);
- Data checks and quality controls (e.g. de-duplication for multiple repeat positive samples per patient);
- Previous epidemiological validation study of the laboratory-based automated electronic surveillance method against conventional epidemiological surveillance methods (e.g. conventional case notification-based or questionnaire-based data collection methods for surveillance of a particular disease);
- Replacement of conventional/case-based reporting epidemiological surveillance protocols by automated M2M laboratory-based reporting systems;
- Use of healthcare vocabulary/terminology standards (e.g. Logical Observation Identifiers Names and Codes (LOINC)-controlled terminology, International Statistical Classification of Diseases and Related Health Problems, 10th revision (ICD-10) medical classification list in LIMS-generated data format and the Systematic Nomenclature of Medicine Clinical Terms (SNOMED-CT)); and
- Plans, if any, to use automated digital laboratory information reporting systems in the near future for public health purposes.

Box **EU priority indicator antimicrobial-resistant pathogens [19] and antiviral-resistant pathogens in humans under EU/EEA surveillance**

- Meticillin-resistant *Staphylococcus aureus*
- 3rd-generation cephalosporin-resistant *Escherichia coli*
- *Klebsiella pneumoniae* resistant to aminoglycosides, fluoroquinolones and 3rd-generation cephalosporins
- *Streptococcus pneumoniae* resistant to penicillin and macrolides
- *Klebsiella pneumoniae* resistant to carbapenems
- HIV resistant to anti-retroviral agents and influenza virus resistant to neuraminidase inhibitors

EU/EEA: European Union/European Economic Area.

### Definitions used

#### Laboratory information management system

A LIMS is a software system developed to support laboratory operations including results communication. *‘This software system can track specimens and workflows, aggregate data for research or business intelligence purposes, and ensure laboratory operations are compliant with various standards and regulations’* [20].

#### Machine-to-machine communication

*‘Any technology that enables networked devices to exchange information and perform actions without the* *manual assistance of humans’* [21]. This automated communication follows an application programming interface (API), using a set of clearly defined methods of electronic communication among various IT components.

#### Automated machine-to-machine laboratory data reporting

For this study, this is defined as the direct, automated M2M upload of reportable disease laboratory data from clinical LIMS to the national communicable disease surveillance system. This definition is similar to that of Electronic Laboratory Reporting (ELR) used in the United States (US) [22]. It should be noted that the automated transmission may need prior verification/signed authorisation by the clinical laboratory director, in particular where the transferred data relate to mandatory disease notification by a medical doctor.

#### Manual laboratory data reporting

As defined for this study, this includes any other mode of laboratory data reporting, including paper-based reporting by postal mail, facsimile or email; manual reporting by entering data into web-based questionnaire forms and manual file extraction; and upload via the Internet to a central webpage or online database.

### Data collection, validation and analysis

The survey was distributed by email on 25 October 2018 to the NMFPs of 28 EU countries and two EEA countries (Liechtenstein was not included), keeping in copy the National Coordinators of national public health authorities to ensure the best informed respondent in each EU/EEA country was selected to complete the survey. The survey collected information on the 2018 capabilities and capacities of the countries, and was open until 10 December 2018. A report with the preliminary results of the survey was shared with participants on 18 December for their review, and the survey tool was reopened until July 2019 after the results of a preliminary analysis for national data verification and completion of reporting gaps. All questionnaires containing inconsistent or incomplete answers were discussed bilaterally between the survey coordinator and the respondents. Data were thereby completed and validated with the participating experts in the country before inclusion in the final analysis. Data completeness was calculated as a percentage of reported data for each question. Data are presented using descriptive summary statistics.

### Ethical statement

For this survey, we did not seek any ethical review as no personal data were collected. The release of the manuscript including anonymised interview data from the national experts has been approved by all authors.

## Results

### Response rate and data completeness

Each of the 30 EU/EEA countries provided a complete response the survey, i.e. all countries responded to all 21 questions. There were only four ‘I do not know’ replies, by one country each to a different question.

### Use of laboratory information management systems for data reporting from clinical diagnostic laboratories to clinicians

In 2018, a LIMS was used in 28 of the countries by all (n = 9 countries), most (n = 14 countries) or some (n = 5 countries) clinical diagnostic laboratories to manage and report laboratory test results to clinicians. In Bulgaria and Latvia, clinical diagnostic laboratories did not use any LIMS.

### Mode of laboratory data reporting from clinical laboratories to national surveillance databases

**Figure 1:**
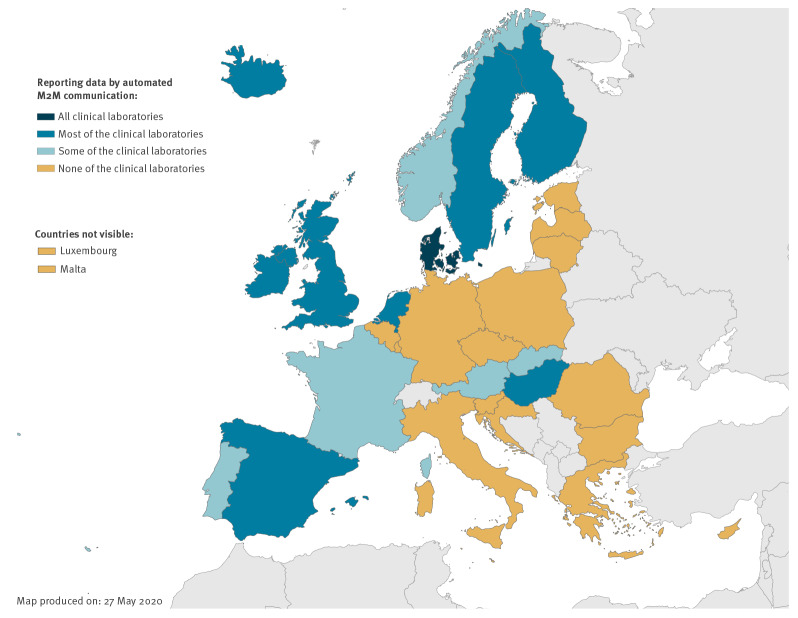
Clinical laboratories reporting data by automated machine-to-machine communication to national surveillance databases, 30 EU/EEA countries, December 2018

In 16 countries, clinical diagnostic laboratories only reported data manually to national surveillance databases, while in 14 countries, all (n = 1 country), most (n = 8 countries) or some (n = 5 countries) clinical diagnostic laboratories reported digital data automatically M2M from their LIMS to national databases (Figure 1). In Denmark, all clinical laboratories used automated M2M reporting to the national surveillance databases without any manual intervention. In another 13 countries, either most diagnostic laboratories (Finland, Hungary, Iceland, Ireland, the Netherlands, Spain, Sweden and the UK) or some diagnostic laboratories (Austria, France, Norway, Portugal and Slovakia) reported data by automated M2M communication to national databases. (Figure 1). Diverse automated reporting systems were used by laboratories in six countries whereas a single, generic reporting system for all diseases was available in eight countries. The majority of the countries (n = 25) had plans to start using or further expand their automated laboratory information reporting systems in the near future for public health purposes.

EU/EEA: European Union/European Economic Area; M2M: machine-to-machine.

The question in the questionnaire was categorical, with response options ‘all’, ‘most’, ‘some’ or ‘none’.

### Type of laboratories reporting by automated machine-to-machine communication to national databases

Among the 14 countries with automated M2M laboratory data reporting to national surveillance databases, diagnostic laboratories from the public sector reported on an automated basis to national databases in 12 countries, commercial diagnostic laboratories in 10 countries and academic hospital laboratories in eight countries. National reference laboratories also reported on an automated basis to national databases in 10 countries.

### Laboratory data reporting for EU-notifiable diseases

**Figure 2:**
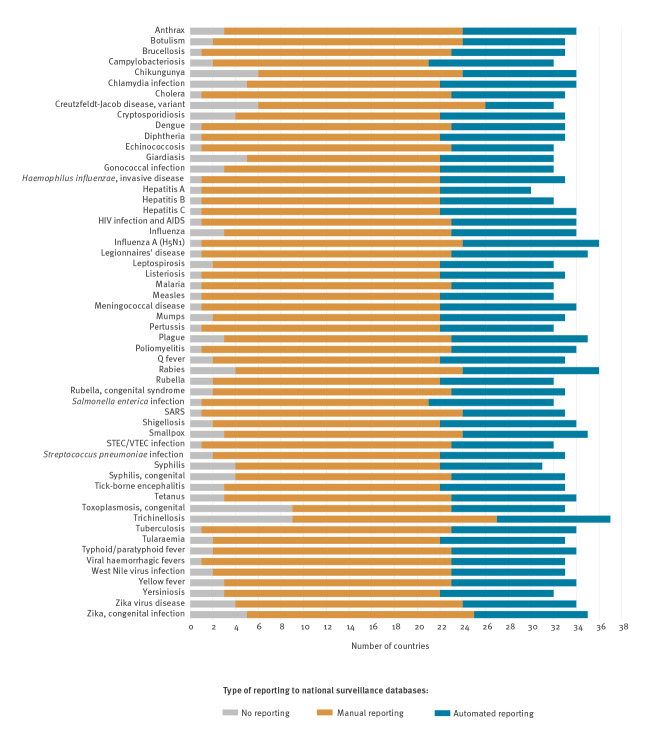
Countries reporting EU-notifiable disease data from clinical diagnostic laboratories to national surveillance databases, by disease and reporting method^a^, 30 EU/EEA countries, December 2018

Figure 2 shows the proportion of EU/EEA countries where EU-notifiable disease related laboratory data were reported to national surveillance databases in 2018 by disease and reporting method. Laboratory data on some parasitic diseases, e.g. trichinellosis and congenital toxoplasmosis, and/or rare diseases, e.g. variant Creutzfeldt-Jacob disease (vCJD), chikungunya, were least frequently reported by laboratories using either manual or automated methods. Overall 23 of 56 notifiable diseases were reported from the laboratory to a national database in all but one country. Among the diseases reported to national surveillance databases, the number of countries reporting laboratory data automated was six for vCJD and ranged from nine to 12 for the other notifiable diseases.

EU/EEA: European Union/European Economic Area; SARS: severe acute respiratory syndrome; STEC/VTEC: Shiga-toxin/verocytotoxin-producing *Escherichia coli*.

^a^ Multiple answers possible.

### Reporting on indicator antimicrobial-resistant pathogens and antibiotic susceptibility data

**Figure 3:**
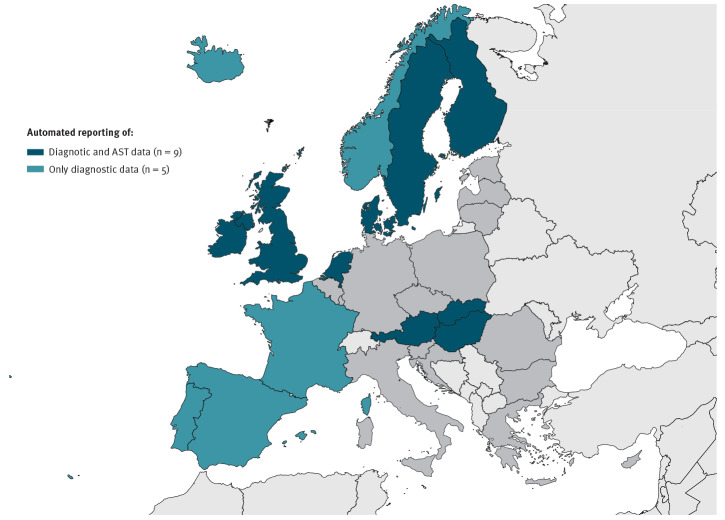
Automated reporting of clinical laboratory data (diagnostic and antimicrobial susceptibility testing data^a^) to national surveillance databases, 14 EU/EEA countries with automated laboratory-based surveillance capability, December 2018

Data on some or all the EU priority indicator antimicrobial-resistant pathogens were reported from clinical laboratories by automated M2M communication to the national surveillance level in nine of 14 countries with automated laboratory-based surveillance capability (Figure 3).

AST: antibiotic susceptibility testing; EU/EEA: European Union/European Economic Area.

^a^ Diagnostic and susceptibility data refer to all or some EU priority indicator antimicrobial-resistant pathogens.

**Figure 4:**
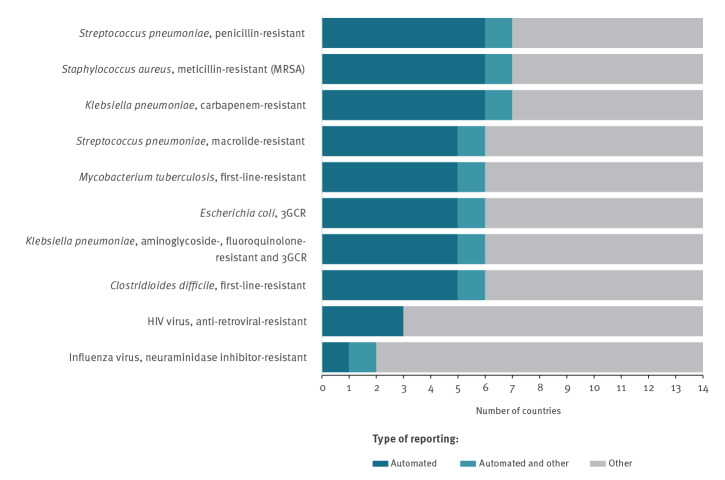
Modes clinical diagnostic laboratories use to report EU priority indicator antimicrobial-resistant pathogens^a^ to national surveillance databases, 14 EU/EEA countries with automated laboratory-based surveillance capability, December 2018

Antibiotic susceptibility testing (AST) results were reported in those nine countries by automated M2M communication in a qualitative format (categorised as ‘resistant’, ‘intermediate’, ‘susceptible’) according to European Committee on Antimicrobial Susceptibility Testing (EUCAST) 2018 version 8.0 definitions and clinical breakpoints [23]. In addition, five of these countries automatically also reported AST data in quantitative formats, like the disk diffusion zone size or the minimum inhibitory concentration (MIC). Positive results for detection and identification of specific AMR mechanism/determinants, e.g. extended-spectrum beta-lactamases, were further reported automatically in seven of these countries (Figure 4). For bacterial pathogens, approximately half of the 14 countries reported AMR data by automated M2M communication, while resistance to viral pathogens such as HIV and influenza virus was predominantly reported manually.

EU/EEA: European Union/European Economic Area; 3GCR: third generation cephalosporin-resistant.

^a^ See [19].

### Data use for cluster detection and early warning at national level

In all but one country with automated M2M laboratory data reporting, the daily or weekly transmitted data were used at the national level for continuous or frequent cluster event detection analysis and early warning for public health purpose. Five countries analysed these data for all the pathogens reported to the national system, while the remaining eight countries did this only for selected diseases. The latter included respiratory diseases (influenza, *Mycoplasma pneumoniae* infection, respiratory syncytial virus infection), food-borne diseases (salmonellosis, campylobacteriosis, shigellosis, listeriosis), sexually transmitted diseases (syphilis, gonorrhoea, chlamydia infection, viral hepatitis, HIV infection), multidrug-resistant pathogens (multidrug-resistant tuberculosis, meticillin-resistant *Staphylococcus aureus* (MRSA)) and arbovirus infections/viral haemorrhagic fevers.

### Type of data reported by automated machine-to-machine communication

The type of data reported automatically by M2M from clinical laboratories’ LIMS to national surveillance systems in 14 EU/EEA countries is described in the Table. The type of clinical specimens tested was more systematically reported than the type of diagnostic tests performed. Positive test results were more frequently reported than negative test results. Denominator data on the total number of diagnostic tests performed and the number of clinical specimen tested were reported by laboratories in less than half of these countries. Basic patient demographic data (age and sex) were reported in all countries whereas data on patient place of residence were also reported in most countries. Patient clinical and epidemiological data were reported in approximately half of these 14 countries on the vaccination status (immunisation history), and the healthcare or community association of the diagnosed infection. Meanwhile, nine of 14 countries reported data from LIMS on patient history of recent travel outside their country of residence.

**Table:** Type of data reported automatically to national surveillance databases, 14 EU/EEA countries with automated laboratory-based surveillance capability, December 2018

Data type	Number of countries reporting on automated basis (n = 14)
**Laboratory test data**	
Type of clinical specimen tested	13
Type of diagnostic test performed	10
Number of clinical specimens tested	6
Number of diagnostic tests performed	3
Positive diagnostic test results	12
Quantitative diagnostic test results (e.g. serology results, NAT results)	6
Negative diagnostic test results	4
**Patient demographic data**	
Age	14
Sex	14
Place of residence	13
**Clinical and epidemiological data**	
Clinical diagnosis	8
Underlying disease	4
Treatments	3
Vaccination status	7
Date of onset of disease	9
Community/healthcare-associated disease	6
Travel history	9

EU/EEA: European Union/European Economic Area; NAT: nucleic acid amplification testing.

### Semantic coding and data cross-linkage

Regarding the interoperability formats of messages from M2M laboratory reporting, standard healthcare vocabulary or controlled coding terminology was used in seven countries. The coding standards include ICD-10 in three countries and SNOMED-CT in one. The microbiological diagnostic test codes comparable to the LOINC was used in two countries. Two countries did not specify the vocabulary used.

In seven of the 14 countries using automated M2M reporting, the reported laboratory data were automatically linked to case-based notified epidemiological data. In six countries, the laboratory data were also automatically linked to other databases. Personal data protection issues and information governance controls were mentioned as main obstacles in the countries not cross-linking the data.

### Data quality and epidemiological validation of electronically reported laboratory data for surveillance

National surveillance systems allowed for the de-duplication of positive samples per patient in all 14 countries reporting data M2M. In six countries, the accuracy of the laboratory-based automated electronic surveillance methods was reported to have been epidemiologically validated against standard epidemiological surveillance methods such as manual case notification or questionnaire-based data collection methods for disease surveillance. In five of these 14 countries, automated M2M laboratory reporting systems had replaced some of the conventional epidemiological surveillance protocols using case-based reporting.

### Obstacles to automated laboratory reporting

The two most frequent reasons why clinical laboratories were not automatically reporting data to national surveillance databases by M2M communication were a lack of technical IT support (n = 17 countries) and a lack of financial compensation for data reporting (n = 15 countries). In seven countries, there was no legal basis/obligation for automated laboratory data reporting and in four countries, personal data protection was cited as a major obstacle for automated reporting. Further reasons mentioned included the non-relevance of such data for surveillance, data ownership, the diversity of LIMS within a country, and lack of standardisation for coding dictionaries, computer systems and computer security systems.

## Discussion

This report on current EU/EEA practices and obstacles at the national level offers a basis for surveillance system improvement and efficiency gains through automated laboratory data reporting. It further provides perspective for future automated EU-wide laboratory based surveillance.

Timely and complete reporting of diagnostic microbiology test results to different stakeholders is essential for effective medical and public health infection management.

Within the EU/EEA, the exact number of clinical microbiology laboratories by country is not known. According to a membership survey by the European Society of Clinical Microbiology and Infectious Diseases (ESCMID), the number of microbiological service providers in Europe ranged widely from four to 69 laboratories per 10 million inhabitants in 2011 [24].

ECDC published an overview by notifiable disease of the national surveillance systems operating in 2016, indicating differences and commonalities across the EU/EEA [25]. However, individual countries’ use of standardised vocabulary and data constraints were not described. The present survey is the first to map the status of automation of microbiology data reporting from clinical LIMS to the national public health level in the EU/EEA.

In 2018, about half of the EU/EEA countries had electronic reporting systems in place for automated surveillance of notifiable infectious diseases by M2M data transfer from clinical laboratories to the national surveillance databases. This compares with a progress report from US Centers for Disease Control and Prevention (CDC) indicating that 31% of the 10,600 reporting clinical laboratories from 55 of 57 jurisdictions in the US were reporting data electronically to the CDC in 2014 [26]. This process had been supported with national funding since 2010 through the Epidemiology and Laboratory Capacity for Infectious Diseases cooperative agreement. The proportion of electronic laboratory reporting of surveillance data across the EU/EEA countries varied by disease, as was the case in the US [26]. In 2018, the CDC reported that by May 2018, state health departments in the US received 80% of laboratory reports electronically [27].

The promotion of interoperability standards between the different types of computer systems is key to effective electronic exchange of information between different institutions. In the context of laboratory-based surveillance, this includes use of a structured message syntax, such as Health Level 7 International (HL7) and of semantic standards for coding laboratory tests and diagnostic results as well as clinical observations. A concerted adoption of data standards and their gradual implementation by reporting laboratories coupled with central data processing algorithms has been implemented for national laboratory-based electronic surveillance of communicable diseases in both large countries like the US [28,29] and small countries like Denmark [3,11,12,30]. Universal adoption occurred faster in Denmark than in the US, where considerably more clinical laboratories had to adapt their LIMS to connect to the national reporting system [29,30].

Likewise, in EU/EEA countries, the standardisation of health information systems and technologies needed for national implementation of automated M2M LIMS data reporting for communicable diseases is especially challenging in large countries with decentralised healthcare systems. This is further compounded by the different health system structures, e.g. mix of public/private laboratory and healthcare service providers. The lack of a legal basis for automated laboratory-based surveillance in many countries makes clinical laboratory participation voluntary and requires IT services to adapt LIMS, which involves investing time and money outside their core business. As reported in this survey, the main obstacles to automated laboratory-based surveillance were insufficient IT support, lack of financial incentive followed by lack of legal mandate for automated data reporting. From the legal standpoint, it is important to integrate automated laboratory data reporting workflows with notifications by authorised health personnel for diseases or conditions under national statutory surveillance [30]. In addition, health data sharing with public health authorities must guarantee the personal data protection safeguards [31].

For two decades, the US has invested in laboratory electronic data transmission using a standard reporting process and format in collaboration with clinical laboratories, LIMS software developers and vendors, and public health agencies [28,29]. Equally, close partnership between all stakeholders, including clinical microbiologists, suppliers of LIMS, clinical users, public health epidemiologists and political decision-makers has been key to the success of the Danish Microbiology Database (MiBa) project, as well as its further application to monitor healthcare-associated infections and vaccine effectiveness through cross-linkage with other public registries such as administrative healthcare databases and national vaccine registries [30]. The MiBa receives copies of positive and negative test reports from all departments of clinical microbiology, and provides data in real-time for the surveillance of communicable diseases, thereby enabling rapid detection of outbreaks and timely analysis of trends [30,32]. After extensive validation studies for completeness and accuracy against conventional surveillance, the MiBa has replaced manual data reporting in Denmark for a number of diseases under surveillance, including influenza [32], pertussis [30], *Clostridiodes difficile* infection [12], Lyme neuroborreliosis [3] and healthcare-associated infections [11].

It is encouraging that in the present survey, 13 EU/EEA countries reported that automated laboratory data transmission was made operational for cluster detection analysis and early warning at the national level. Notably, automated reporting had replaced conventional surveillance after epidemiological validation in six countries. These findings provide further evidence of the added value of this novel surveillance approach for gains in efficiency and public health effectiveness, as also reported for the detection of hospital outbreaks [8], hepatitis A contact prophylaxis [33] and alerting of antimicrobial resistance outbreaks [7].

This study has several limitations. The self-reporting nature of the survey makes data subject to subjective interpretation by the national experts collecting the information. Possible variance in inter-reporter understanding of the survey terms was to some degree reduced by piloting the questionnaire and developing a glossary of terms and definitions via a series of discussions held individually and in the National Focal Points Forum. It was also reduced to some degree by performing a bilateral validation of each national dataset by the investigators and survey responders. As some questions, such as the use of laboratory data for early warning at the national public health level and frequency of cluster detection analysis, were only addressed to countries performing automated LIMS data reporting, we do not know to what degree this public health output differed in countries using manual data reporting.

Looking to the future, the ECDC microbiology strategy envisions that in 2022, at least 90% of EU/EEA countries will be using real-time, automated M2M reporting of clinical laboratory data to national surveillance programmes [34]. Achieving this will require new policies and health system investments in many of these countries. However, such an advanced European-wide e-surveillance framework would open up further opportunity for international reporting, and the timely detection and management of cross-border health threats. The feasibility of translating national laboratory-based electronic surveillance approaches to the European surveillance level will be explored jointly by ECDC and the European Commission as part of broader EU digital health support programmes.

## ECDC National Focal Points laboratory e-reporting survey group

Franz Allerberger, Austrian Agency for Health and Food Safety (AGES), Vienna, Austria

Yves Dupont, Sciensano, Brussels, Belgium

Steven Van Gucht, Sciensano, Brussels, Belgium

Sophie Quoilin, Sciensano, Brussels, Belgium

Iva Christova, National Centre of Infectious and Parasitic Diseases, Sofia, Bulgaria

Blazenka Hunjak, Croatian Institute of Public Health, Zagreb, Croatia

Christos Karagiannis, Nicosia General Hospital, Stovolos, Nicosia, Cyprus

Pavla Křížová, National Institute of Public Health, Prague, Czechia

Jitka Částková, National Institute of Public Health, Prague, Czechia

Eva Møller Nielsen, Statens Serum Institut, Copenhagen, Denmark

Jonas Kähler, Statens Serum Institut, Copenhagen, Denmark

Rita Peetso, Health Board, Tallinn, Estonia

Saara Salmenlinna, National Institute for Health and Welfare (THL), Helsinki, Finland

Teemu Möttönen, National Institute for Health and Welfare (THL), Helsinki, Finland

Bruno Coignard, Santé Publique France, Saint-Maurice, France

Michaela Diercke, Robert Koch Institute, Berlin, Germany

Alkiviadis Vatopoulos, National School of Public Health, Athens, Greece

Ákos Tóth, National Public Health Center, Budapest, Hungary

Karl Gustaf Kristinsson, Landspitali University Hospital, Reykjavik, Iceland

Eleanor McNamara, Public Health Laboratory, Dublin, Ireland

Annalisa Pantosi, Istituto Superiore di Sanità (ISS), Rome, Italy

Violeta Mavcutko, Centre for Disease Prevention and Control, Riga, Latvia

Algirdas Griškevičius, National Public Health Surveillance Laboratory, Vilnius, Lithuania

Joël Mossong, Laboratoire National de Santé (National Health Laboratory), Luxembourg

Christopher Barbara, Mater Dei Hospital, Msida, Malta

Titia Kortbeek, Rijksinstituut voor Volksgezondheid en Milieu (RIVM), Bilthoven, Netherlands

Dominique Caugant, Norwegian Institute of Public Health, Oslo, Norway

Ulf Dahle, Norwegian Institute of Public Health, Oslo, Norway

Line Vold, Norwegian Institute of Public Health, Oslo, Norway

Anna Skoczyńska, National Medicines Institute, Warsaw, Poland

Jorge Machado, National Institute of Health Dr Ricardo Jorge (INSA), Lisbon, Portugal

Gabriel Ionescu, ‘Cantacuzino’ National Institute, Bucharest, Romania

Lucia Madarova, Regional Public Health Authority, Banska Bystrica, Slovakia

Mária Avdičová, Regional Public Health Authority, Banska Bystrica, Slovakia

Metka Paragi, National Institute of Public Health, Ljubljana, Slovenia

Julio Moreno Vazquez, Instituto de Salud Carlos III, Majadahonda, Spain

Karin Tegmark Wisell, Folkhälsomyndigheten, Solna, Sweden

Maria Zambon, Public Health England, London, United Kingdom
